# Down-regulation of oxidative phosphorylation in the liver by expression of the ATPase inhibitory factor 1 induces a tumor-promoter metabolic state

**DOI:** 10.18632/oncotarget.6357

**Published:** 2015-11-22

**Authors:** Fulvio Santacatterina, Laura Sánchez-Cenizo, Laura Formentini, Maysa A. Mobasher, Estela Casas, Carlos B. Rueda, Inmaculada Martínez-Reyes, Cristina Núñez de Arenas, Javier García-Bermúdez, Juan M. Zapata, María Sánchez-Aragó, Jorgina Satrústegui, Ángela M. Valverde, José M. Cuezva

**Affiliations:** ^1^ Departamento de Biología Molecular, Centro de Biología Molecular Severo Ochoa, CSIC-UAM, Madrid, Spain; ^2^ Centro de Investigación Biomédica en Red de Enfermedades Raras (CIBERER), Madrid, Spain; ^3^ Centro de Investigación Hospital 12 de Octubre, ISCIII, Madrid, Spain; ^4^ Instituto de Investigaciones Biomédicas Alberto Sols, CSIC-UAM, Madrid, Spain; ^5^ Centro de Investigación Biomédica en Red de Diabetes y Enfermedades Metabólicas Asociadas (CIBERDEM), Madrid, Spain; ^6^ Instituto de Investigación Sanitaria Fundación Jiménez Díaz (IIS-FJD), Madrid, Spain

**Keywords:** ATPase inhibitory factor 1, cancer, energy metabolism, mitochondria, reactive oxygen species

## Abstract

The ATPase Inhibitory Factor 1 (IF1) is an inhibitor of the mitochondrial H^+^-ATP synthase that regulates the activity of both oxidative phosphorylation (OXPHOS) and cell death. Here, we have developed transgenic Tet-On and Tet-Off mice that express a mutant active form of hIF1 in the hepatocytes to restrain OXPHOS in the liver to investigate the relevance of mitochondrial activity in hepatocarcinogenesis. The expression of hIF1 promotes the inhibition of OXPHOS in both Tet-On and Tet-Off mouse models and induces a state of metabolic preconditioning guided by the activation of the stress kinases AMPK and p38 MAPK. Expression of the transgene significantly augmented proliferation and apoptotic resistance of carcinoma cells, which contributed to an enhanced diethylnitrosamine-induced liver carcinogenesis. Moreover, the expression of hIF1 also diminished acetaminophen-induced apoptosis, which is unrelated to differences in permeability transition pore opening. Mechanistically, cell survival in hIF1-preconditioned hepatocytes results from a nuclear factor-erythroid 2-related factor (Nrf2)-guided antioxidant response. The results emphasize *in vivo* that a metabolic phenotype with a restrained OXPHOS in the liver is prone to the development of cancer.

## INTRODUCTION

Mitochondria play key roles in cell metabolism and bioenergetics, mediate intracellular signaling by calcium and reactive oxygen species (ROS) and regulate the execution of cell-death [1]. Most of the ATP that is required to maintain cellular activities is synthesized by the mitochondrial H^+^-ATP synthase [2]. Down-regulation of oxidative phosphorylation (OXPHOS) and the concurrent activation of aerobic glycolysis is a hallmark of proliferating cancer cells [1, 3] whereas an increase in oxidative metabolism halts cellular proliferation and tumor progression [1, 4, 5]. The activity of OXPHOS is required for the execution of cell death [1, 6] and in particular, the ATP synthase is needed for the execution of apoptosis [7] as recently demonstrated in neurons *in vivo* [8]. In fact, the ATP synthase is a critical component in the permeabilization of the inner mitochondrial membrane to low molecular weight solutes, i.e., in the opening of the permeability transition pore (PTP) [9-11]. Not surprisingly, inhibition of the ATP synthase is involved in lifespan extension [12, 13] illustrating the relevance of this protein complex in aging and age-related diseases.

In mitochondria, futile ATP hydrolysis by the ATP synthase is inhibited by the ATPase Inhibitory Factor 1 (IF1) a small nuclear-encoded protein that reversibly binds to the enzyme [2]. Data obtained in cancer [14, 15], in stem cells [16], and in a mouse model over-expressing an active form of IF1 in neurons[8], support that *in vivo* IF1 also inhibits the synthase activity of the ATP synthase. The IF1-mediated inhibition of the ATP synthase prevents cell death [8, 14, 17]. Remarkably, IF1 is highly over-expressed in human carcinomas [14, 15, 18]. In hepatocarcinomas the over-expression of IF1 favors angiogenesis and metastasis [19].

Herein, we have questioned: What is the relevance of a metabolic phenotype with a restrained OXPHOS in cancer onset and progression *in vivo?* To that aim we have generated transgenic mice that express a mutant active form of human IF1 (hIF1) under a tetracycline regulated promoter in hepatocytes. We show that expression of the transgene promotes inhibition of OXPHOS and a higher susceptibility to diethyl-nitrosamine (DEN)-induced carcinogenesis. Mechanistically, an enhanced carcinogenesis in hepatocytes of hIF1 expressing mice involves an enhanced proliferation and the down-regulation of the potential to execute cell death as further illustrated *in vivo* in a model of acetaminophen (APAP)-induced hepatotoxic damage. Cell survival in hIF1 expressing hepatocytes is not mediated by differential regulation of PTP opening in response to APAP treatment but by the induction of a nuclear factor-erythroid 2-related factor (Nrf2)-guided antioxidant response. These findings emphasize that metabolic preconditioning by restraining OXPHOS is deleterious in the context of liver cancer because it limits cell death favoring the progression of oncogenic events.

## RESULTS

### *In vivo* IF1-mediated inhibition of OXPHOS in the liver of Tet-Off mice

**Figure 1:**
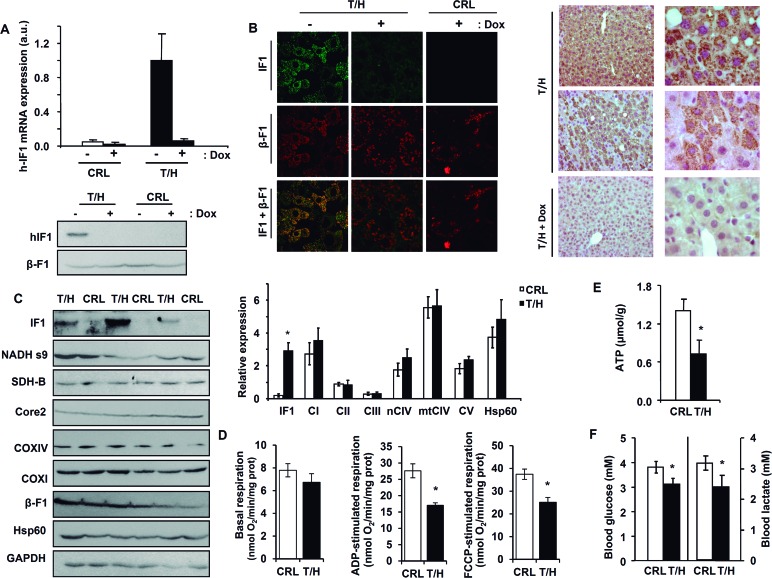
OXPHOS is inhibited in the liver of mice expressing hIF1 Only the double transgenic animals (T/H) expressed hIF1 in the absence of Doxycycline (Dox) administration. **A**. hIF1 mRNA expression in the liver of control (CRL) and T/H mice. Blot of hIF1 (12 kDa) and β-F1-ATPase (β-F1) in T/H and CRL mice. **B**. Double-immunofluorescence microscopy (63X) of liver from CRL and T/H mice stained with IF1 (green) and β-F1-ATPase (red) antibodies. Merged images (IF1+β-F1). Immunohistochemistry for hIF1 in the liver of T/H mice at 20X and 63X magnification. **C**. Blots of hIF1, complex I (NADH s9), complex II (SDH-B), complex III (Core2), complex IV (COXI and COX IV) and complex V (β-F1), Hsp60 and GAPDH in liver extracts of CRL and T/H mice. Histograms to the right show the relative expression of each protein normalized to GAPDH expression. **D**. Polarographic determination of basal, ADP-stimulated and FCCP-stimulated respiration in isolated liver mitochondria of CRL and T/H mice. The results are the mean ± SEM of 8 mice per group. **E**. ATP concentration in liver extracts of CRL and T/H mice. The results shown are the mean ± SEM of 5 mice per group. **F**. Blood glucose and lactate concentrations of fasted CRL and T/H mice. Histograms are the mean ± SEM of 12-14 mice per group. (C-F) *, *p* < 0.05 when compared to control by Student's t test. See also Figures S1 and S2.

Breeding of mice expressing the tTA transactivator in liver with transgenic mice containing the human IF1-H49K transgene (hIF1) resulted in double transgenic mice (T/H). The double transgenic animals (T/H) expressed hIF1 in the absence of Doxycycline (Dox) administration as revealed by the presence of hIF1 mRNA and protein levels in their livers (Figure 1A). Expression of hIF1 is restricted to mitochondria of hepatocytes (Figure 1B) and negatively regulated by the administration of Dox as revealed by qPCR and western blotting (Figure 1A), confocal microscopy (Figure 1B, panels to the left) and immunohistochemistry (Figure 1B, panels to the right). The expression of hIF1 had no impact on the expression level of relevant mitochondrial proteins of different OXPHOS complexes (Figure 1C). Isolated liver mitochondria from adult T/H mice revealed that both the ADP-stimulated respiration and the maximum respiratory rates were significantly reduced when compared to littermate controls (Figure 1D). In addition, liver ATP concentrations were diminished in T/H mice (Figure 1E). An overnight fast of adult Tet-Off T/H mice promoted a significant reduction in blood glucose and lactate concentrations (Figure 1F).

### *In vivo* IF1-mediated inhibition of OXPHOS in the liver of Tet-On mice

Breeding of mice expressing the rtTA-Adv transactivator in liver with transgenic mice containing the human IF1-H49K transgene (hIF1) (Supplemental Figure S1A) resulted in double transgenic mice (T/H) (Supplemental Figure S1B) able to express hIF1 in the liver only in response to the administration of doxycycline (Dox) (Supplemental Figure S1B). The expression level of hIF1 varied among different T/H mice (Supplemental Figure S1B) and was higher than that of the endogenous IF1 present in mouse liver of control littermates (Supplemental Figure S1C). Expression of hIF1 was preferentially concentrated in perivenous hepatocytes (Supplemental Figure S1D) and had no impact on the expression level of relevant mitochondrial proteins of different OXPHOS complexes (Supplemental Figure S1E). T/H mice showed no differences in weight, life span and cage behavior when compared to controls after one year of follow up. However, and consistent with the inhibitory role of IF1 on the activity of the H^+^-ATP synthase we observed that both the basal and the ADP-stimulated respiration in isolated mitochondria from adult livers of T/H mice was significantly reduced when compared to controls (Supplemental Figure S1F). A significant decrease in the maximum respiratory rate was also noted in mitochondria of T/H mice (Supplemental Figure S1F). Overall, these findings indicate that expression of hIF1 is partially arresting both respiration and ADP phosphorylation in liver mitochondria.

In agreement with a partial energy deficit in the liver of T/H mice we observed: (i) reduced ATP and increased AMP contents in their livers (Supplemental Figure S2A), (ii) a significant increase in the AMP/ATP ratio (Supplemental Figure S2A) and (iii) the activation by phosphorylation of the metabolic stress kinase AMPK (Supplemental Figure S2B), when compared to liver of controls. The pyruvate content was also diminished in the liver of T/H mice (Supplemental Figure S2C). Other liver organic acids showed no relevant differences (Supplemental Figure S2C). Blood glucose and lactate concentrations were not significantly different between fed T/H and control mice (Supplemental Figure S2D). However, an overnight fast promoted a marked hypoglycaemia (Supplemental Figure S2E) and hypolactatemia (Supplemental Figure S2E) in T/H mice. The liver of 15 day-old T/H neonates revealed a significant induction of mitochondrial superoxide dismutase (SOD2) (Supplemental Figure S2F). Functionally, induction of SOD2 in liver of T/H mice was expressed by less carbonylation of some cellular proteins when compared to livers of control mice (Supplemental Figure S2G). These results suggest that a limited OXPHOS by the expression of hIF1 in the liver of T/H mice compromises the energy supply needed for normal activity of gluconeogenesis and eventually favors the utilization of lactate for oxidative purposes in extrahepatic tissues during fasting. Overall, using two different experimental systems to regulate the expression of hIF1 in mouse liver we show that its expression triggers an energy deficit in the hepatocytes by inhibiting the activity of OXPHOS. Dox is known to induce the arrest of mitochondrial protein translation [20, 21], and eventually, to promote the unfolded protein response in mitochondria (UPRmt) [22]. Since we obtained similar findings with a Tef-Off mouse model (Figure 1), we ruled out any role for Dox in triggering metabolic adaptation by unleashing the UPRmt.

### Expression of hIF1 affects the activity and assembly of Complex IV and Complex V

**Figure 2:**
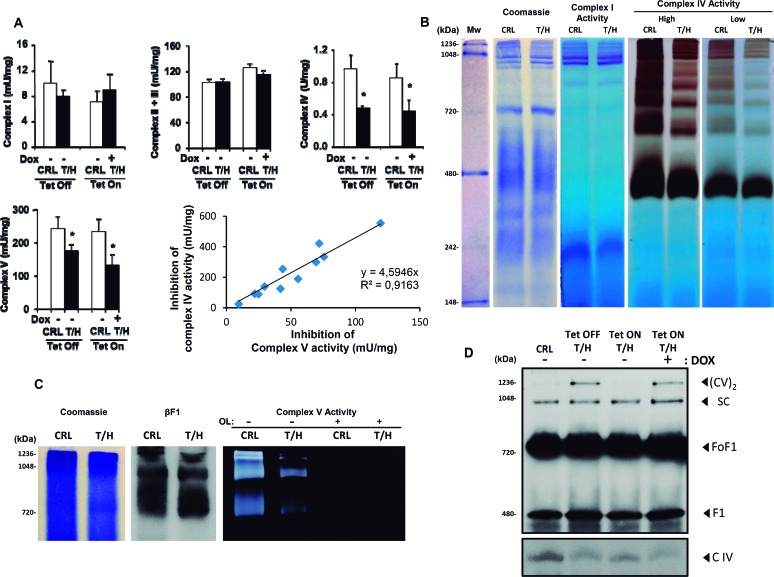
Activities and assembly of OXPHOS complexes in the liver of hIF1 mice **A**. Enzymatic activities of complex I, II+III, IV and V in isolated liver mitochondria of CRL and T/H mice. Histograms are the mean ± SEM of 3 experiments. *, p<0.05 when compared to control by Student's t test. Linear correlation between the inhibition of Complex V activity by hIF1 and the inhibition of Complex IV activity. **B**. BN-PAGE-in-gel-activities of Complex I and Complex IV (low and high exposures). **C**. CN-PAGE immunoblot analysis of β-F1-ATPase (βF1) and CN-PAGE-in-gel-activity of complex V in the presence (+) or absence (−) of oligomycin (OL). **D**. BN-PAGE immunoblot analysis of mitochondrial complexes IV (COX IV) and V (β-F1-ATPase). The migration of dimers of ATP synthase (CV)_2_, supercomplex (SC), ATP synthase (FoF1), F1-ATPase (F1) and complex IV (C IV) is indicated. See also Supplemental Table S1.

Isolated liver mitochondria from both the hIF1 expressing Tet-On and Tet-Off mice revealed reduced activities of Complex IV and V when compared to controls (Figure 2A). In contrast, the activities of Complex I and II+III were not significantly affected by the expression of hIF1 (Figure 2A). Interestingly, a significant correlation between the inhibition of Complex V and Complex IV activities was observed (Figure 2A). In-gel activity of complexes I and IV revealed that whereas the activity of Complex I was not modified in hIF1 expressing Tet-Off mice, the activity of Complex IV and its super-complexes were very much reduced (Figure 2B). A similar study to assess the oligomycin (OL)-sensitive ATP hydrolase activity in super-complexes confirmed that hIF1 expressing mice had reduced ATPase activity despite essentially containing the same amount of the β-catalytic subunit of the complex in ATPase oligomers (Figure 2C).

Western blotting of BN-gels appears to confirm that the assembly of Complex IV was reduced in both mouse models of hIF1 expressing mice because its monomeric form was diminished when compared to that of controls (Figure 2D), what is in agreement with the reduced Complex IV activity observed in these animals (Figure 2A, 2B). Likewise, the expression of hIF1 in the liver of both Tet-On and Tet-Off mice promoted the assembly of a low migrating super-complex that contained complex V and might represent dimers of the ATP synthase ((CV)_2_ in Figure 2D). Trypsin digestion and mass spectrometric analysis of the three upper bands recognized by anti-β-F1-ATPase antibody supported these results and revealed that the major component of the SC band in Figure 2D is Complex I (see Supplemental Table SI).

### A diminished activity of OXPHOS favors hepatocarcinogenesis

**Figure 3:**
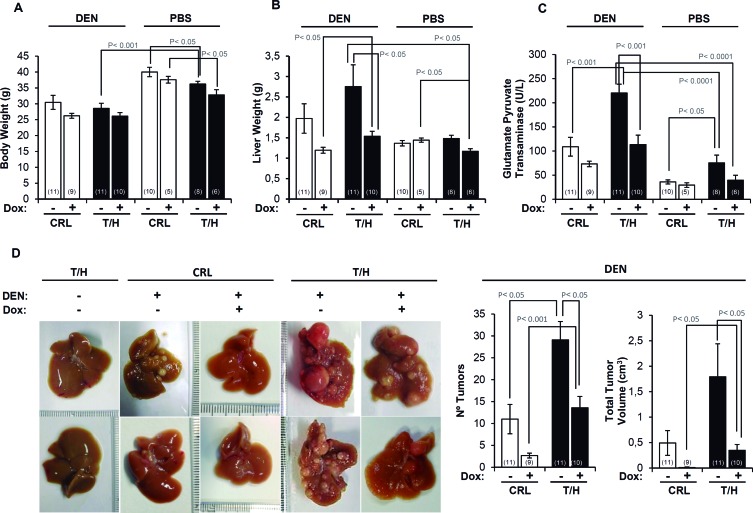
The expression of hIF1 increased DEN-induced hepatocarcinogenesis (HCC) **A**.-**C**. Changes in body weight (A), liver weight (B) and blood GPT (C) in PBS or DEN-treated CRL (open bars) and hIF1 Tet-Off T/H (closed bars) mice, treated in presence (+) or absence (−) of Dox. **D**. CRL and T/H livers of mice treated (+) or not treated (−) with DEN. The effect of Dox administration (+) or not (−) is also shown. The number of tumors and the total tumor volume in DEN-treated CRL (open bars) and T/H (closed bars) mouse livers treated (+) or not (−) with Dox. The number of mice is indicated in parenthesis. Results are means ± SEM. P values by Student's t test are indicated.

**Figure 4:**
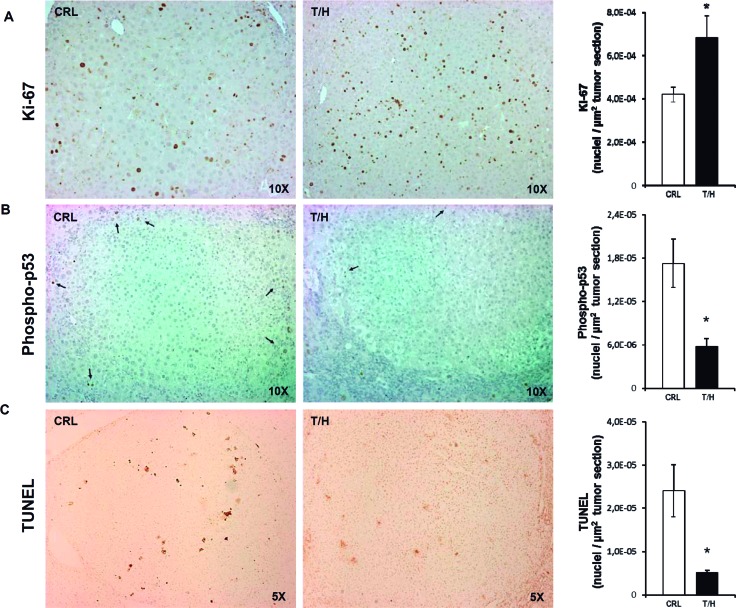
Expression of hIF1 promotes increased proliferation and apoptotic resistance of HCC **A**.-**C**. Liver sections of DEN-treated CRL and hIF1 Tet-Off T/H mice were processed for immunohistochemistry to assess the rates of proliferation (A, Ki-67 staining) and apoptosis (B, phospho-p53 and C, TUNEL) in HCC. Magnification 5x-10x. Bars are the mean ± SEM of 6 mice per group. *, p<0.05 when compared to CRL by Student's *t* test.

A preliminary experiment of DEN-induced hepatocarcinogenesis by single injection of DEN [23] was initially done in a small number of mice (Supplemental Figure S3). Hepatocarcinogenesis was further studied with the long-term DEN administration protocol [24] (Figure 3) because it leads to a higher tumor incidence in a shorter time span [25]. Both protocols provided the same findings (Figure 3 and Supplemental Figure S3). Dox administration slightly reduced the body weight of the animals (Figure 3A). In contrast, the liver weight of DEN-treated mice and especially of those expressing hIF1 was significantly augmented when compared to the rest of the animals (T/H minus Dox in Figure 3B). The increase in liver weight of mice expressing hIF1 was accompanied by a significant progressive increase in blood glutamate-pyruvate transaminase (GPT) activity towards the end of the study (Figure 3C and see Supplemental Figures S3C and S4). Consistent with these observations both the number of tumors and total tumor volume were significantly increased in hIF1 expressing mice (Figure 3D). Dox administration reduced the number of tumors and total tumor volume both in control and hIF1 silenced T/H mice (Figure 3D). The higher tumor burden of mice expressing the transgene resulted from increased rates of proliferation (Figure 4A) and diminished apoptosis (Figure 4B, 4C) as assessed by immunohistochemical analysis of the carcinomas. Overall, these findings indicate *in vivo* the oncogenic role of IF1 and that limiting the activity of OXPHOS in the liver favors cancer onset and progression.

### The mitochondrial phenotype in hepatocarcinogenesis

**Figure 5:**
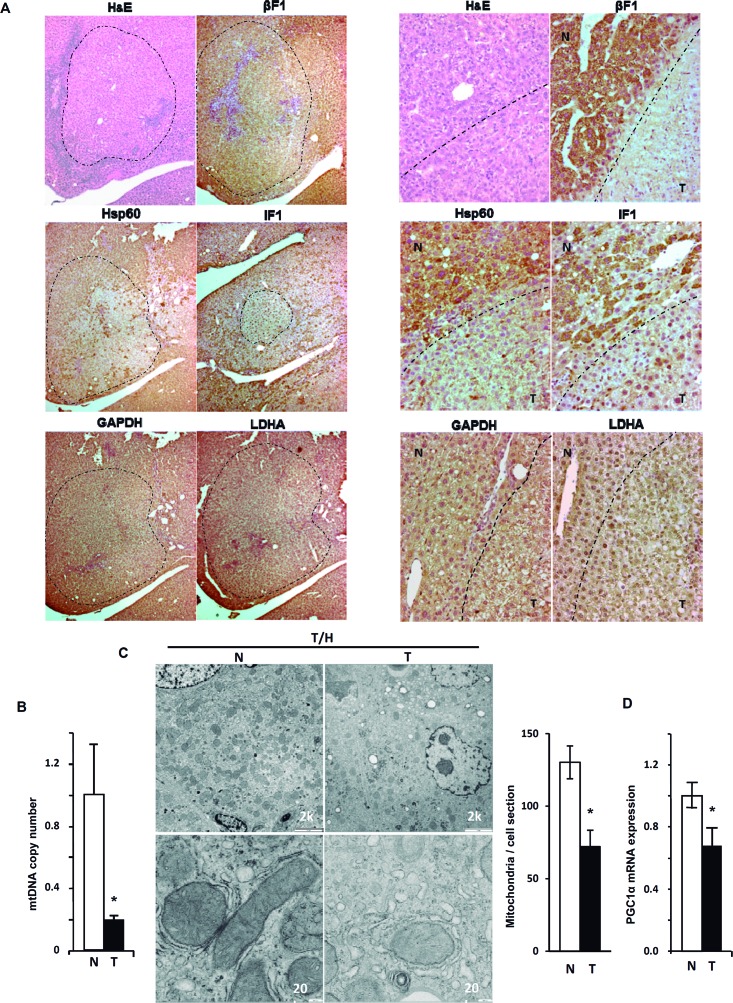
Repression of mitochondrial biogenesis in DEN-induced hepatocarcinogenesis (HCC) **A**. Focal hepatic lesions (magnification 5x) in long-term DEN-treated T/H mice identified by hematoxylin-eosin (H&E) and immunohistochemical staining with the antibodies against β-F1-ATPase (β-F1); Hsp60; hIF1; GAPDH and LDHA in the same localized tumor area (panels to the left). The same study in normal (N, upper left) and tumor (T, lower right) areas at 20X magnification (panels to the right). A dashed line marks the border between tumor and non-tumor areas. **B**. mtDNA copy number in DNA extracted from normal (N) and tumor (T) liver tissue of hIF1 expressing T/H mice. Results are means ± SEM for 6-7 animals. *P < 0.05 when compared with normal by Student's t test. **C**. Representative electron micrographs of normal (N) and tumor (T) tissue of the liver of hIF1 expressing T/H mice. Mitochondria of the hepatocytes in normal liver showed its characteristic high electron density and inner membrane cristae (left panels), whereas mitochondria are barely identifiable in the tumor tissue (right panels). Scale bars: 2k (upper panels); 20k (lower panels). Histograms show the number of mitochondria / cell section in normal (N) and tumor (T) tissue in T/H mice. Results are means ± SEM of five different sections from 3 animals. *P < 0.05 when compared with normal by Student's t test. **D**. Cellular mRNA expression of PGC1α in normal (N) and tumor (T) tissue of hIF1 expressing T/H mice. Results are means ± SEM of 6 experiments.*, *P* < 0.05 when compared with normal by Student's *t* test.

Immunohistochemical, electron microscopy and molecular analysis of the livers revealed that long-term DEN-induced hepatocarcinogenesis in mice largely reproduced the findings on the mitochondrial phenotype previously reported in human hepatocarcinomas [26]. In fact, it was observed that tumor areas in both T/H (Figure 5A) and CRL (Supplemental Figure S5A) were largely devoid of immunostaining for mitochondrial proteins such as β-F1-ATPase, Hsp60 and IF1, suggesting that progression of liver cancer is concurrent with the repression of mitochondrial biogenesis [26]. We observed no relevant differences in the expression of the glycolytic markers GAPDH and LDHA as assessed by inmmunohistochemical (IHC) (Figure 5A and Supplemental Figure S5A). However, since IHC is a qualitative technique we assessed protein expression by western blotting normal and tumor tissue of CRL and T/H mice (Supplemental Figure S5B). The results obtained indicate that carcinogenesis promoted the down-regulation of β-F1-ATPase expression concurrently with the upregulation of GAPDH in the absence of relevant changes in the expression of Hsp60 and LDHA (Supplemental Figure S5C). As a result the bioenergetic signature of the tumors, as assessed by the β-F1-ATPase/GAPDH ratio [26, 27] was significantly diminished when compared to the signature in normal tissues (Supplemental Figure S5C). Overall, we should mention that we did not observe differences in protein expression in the analysis of focal hepatic lesions between control and transgenic animals (Figure 5A and Supplemental Figure S5A).

Determination of mtDNA content in T/H mice revealed a very large reduction of mtDNA in liver tumors when compared to the normal tissue of the same liver (Figure 5B). This finding was further confirmed by assessing the lower cellular content and alteration of mitochondrial structure in these carcinomas by electron microscopy (Figure 5C), confirming the repression of mitochondrial biogenesis in hepatocarcinogenesis [26]. Consistent with the above findings, the expression of the transcriptional co-activator required in mitochondrial biogenesis PGC1α was significantly reduced in mouse carcinomas when compared to normal liver samples (Figure 5D).

Moreover, since DEN-induced hepatocarcinogenesis is an example of inflammation-driven tumorigenesis we also analyzed macrophage recruitment to focal hepatic lesions in CRL and T/H mice using the F4/80 antibody. The results reveal that there are no differences in macrophage recruitment to the tumor areas between control and T/H mice (Supplemental Figure S6).

### Limiting OXPHOS minimizes apoptotic cell-death

**Figure 6:**
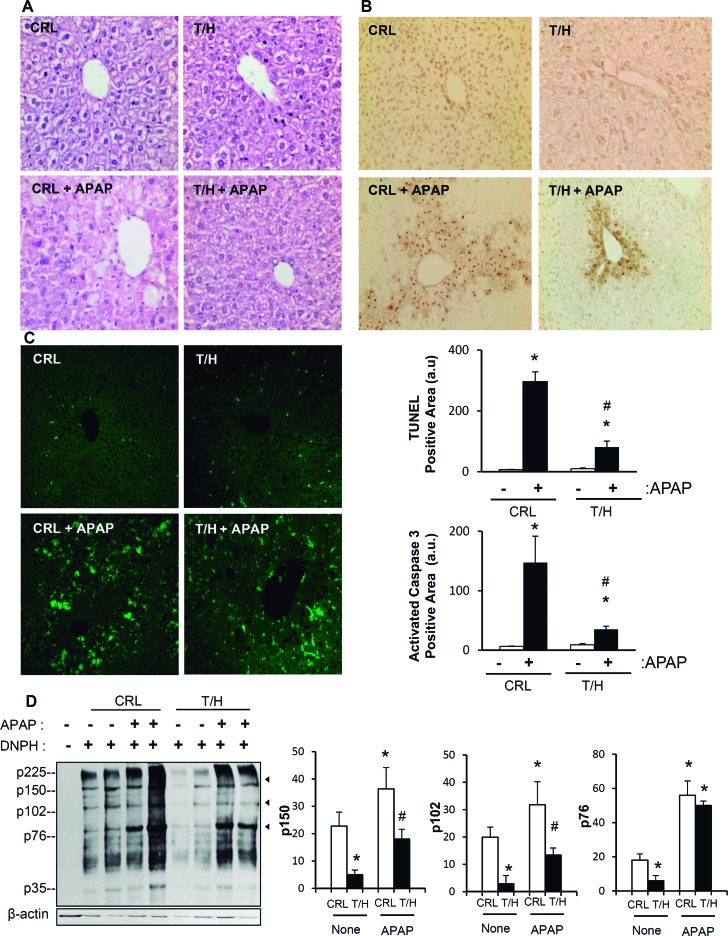
Restraining OXPHOS protects from APAP-induced cell death Control (CRL) and hIF1 (T/H) mice were i.p. injected with APAP (+) or saline (−). Hematoxylin-eosin **A**., TUNEL **B**. and active caspase 3 **C**. staining in liver sections. Magnification 20x. Bars are the mean ± SEM of 6 mice per group. (B,C) *, p<0.05 when compared to non-treated (−) and #, p<0.05 when compared to CRL-APAP treated by Student's t test. **D**. Carbonylation of liver proteins from CRL and hIF1 (T/H) mice treated (+) or non-treated (−) with APAP is shown. The derivation of samples in the presence (+) or absence (−) of DNPH was used for the identification of protein carbonyls. Arrowheads (to the right) identify the migration of the three proteins used in quantification (histograms) of protein carbonylation. The results shown are the mean ± SEM of six CRL and eight T/H mice, respectively. *, *p* < 0.05 when compared to non-treated (−) and #, *p* < 0.05 when compared to CRL-APAP treated by Student's t test.

To provide further mechanistic insight into the main pathway that might participate in the hIF1-mediated stimulation of liver cancer we studied the cell-death response of control and T/H mice to liver induced injury by APAP [28]. APAP overdose promoted a clear centrilobular hepatic cell death at 8h post-administration that was more evident in livers of control than of T/H mice (Figure 6A). Analysis of cell death by terminal deoxynucleotidyl transferase-mediated dUTP nick end labeling (TUNEL) (Figure 6B) and active caspase 3 (Figure 6C) revealed that APAP promoted a higher increase in cell death in control than in T/H mice, strongly supporting that mice with a limited OXPHOS are partially protected from apoptotic cell death. Consistent with previous findings regarding the lower carbonylation observed in liver proteins of T/H mice in the Tet-On model (Supplemental Figure S2G), we noted that the oxidation of liver proteins both under basal conditions and in response to APAP treatment was less pronounced in adult Tet-Off T/H mice (Figure 6D).

### The PTP is not involved in protection against death

**Figure 7:**
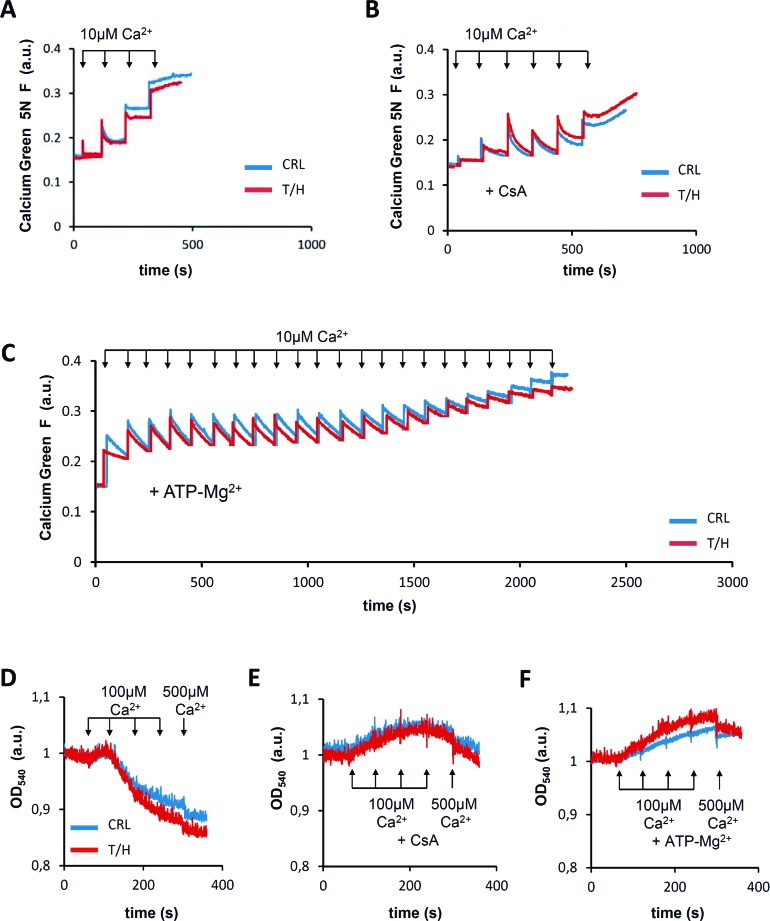
PTP opening and regulation is not affected by hIF1 **A**.-**C**. Calcium green 5N fluorescence in isolated liver mitochondria from control (blue traces) and hIF1 Tet-Off expressing (red traces) mice. Representative experiments show no differences between the calcium retention capacity of liver mitochondria from CRL and T/H mice after the addition of 10μM Ca^2+^ (arrows) in the absence (A) or presence of the PTP blockers 5 μM CsA (B) or 1mM ATP-Mg^2+^ (C). **D**.-**F**. Representative experiments show no differences in Ca^2+^ induced swelling between liver mitochondria derived from CRL and hIF1 expressing T/H mice after the addition of successive 100μM Ca^2+^ (arrows) except the last one which is 500μM Ca^2+^ in the absence (D) or presence of 5 μM CsA (E) or 1mM ATP-Mg^2+^ (F).

Findings in different experimental settings support the role of OXPHOS [1, 6, 29] and of the H^+^-ATP synthase [7, 30] in cell death. Moreover, it has been reported that the PTP is formed by the H^+^-ATP synthase itself [9-11, 31, 32]. To verify the putative implication of the PTP in the differential response to cell death between both mice phenotypes we studied the Ca^2+^-retention capacity (Figure 7A-7C) and Ca^2+^-induced swelling (Figure 7D-7F) of isolated mitochondria from livers of control and hIF1 expressing mice. No relevant differences were observed in any of these parameters between mitochondria of control and T/H mice (Figure 7A & 7D). Moreover, both cyclosporine A (CsA) (Figure 7B & 7E) and ATP-Mg (Figure 7C & 7F), which are known inhibitors of PTP opening [9], exerted the same regulation of the PTP in control and T/H mice. These findings support that inhibition of cell death by IF1 [8, 14, 17] is unrelated with PTP opening. Dimers of the ATP synthase have been shown to be required for PTP formation [11, 32]. Moreover, it has been suggested that IF1 regulates the oligomeric state of the ATP synthase increasing the formation of dimeric ATP synthase complexes and cristae density [33, 34]. Our observation that the expression of hIF1 in the liver of both the Tet-On and Tet-Off mice enhances the assembly of a Complex V super-complex (a putative ATPase dimer) is compatible with a role for IF1 in promoting the dimeric state of the ATP synthase *in vivo*, although in this situation, the IF1-mediated dimerization of the ATP synthase would argue against a role for dimeric ATP synthase as a major element of the PTP [11, 32]. However, we cannot exclude that additional mechanisms that are known to regulate the PTP [9] and the ATP synthase [12, 35, 36] could further participate in the hIF1-mediated inhibition of cell death. In any case, what is evident is that inhibition of the H^+^-ATP synthase mediated either by oligomycin (OL) treatment [7] or by hIF1 expression [8, 14] protects from cell death, further supporting that the activity of the engine of OXPHOS is part of the machinery required for the execution of cell death *in vivo*.

### hIF1 expressing mice are protected against oxidative stress

**Figure 8:**
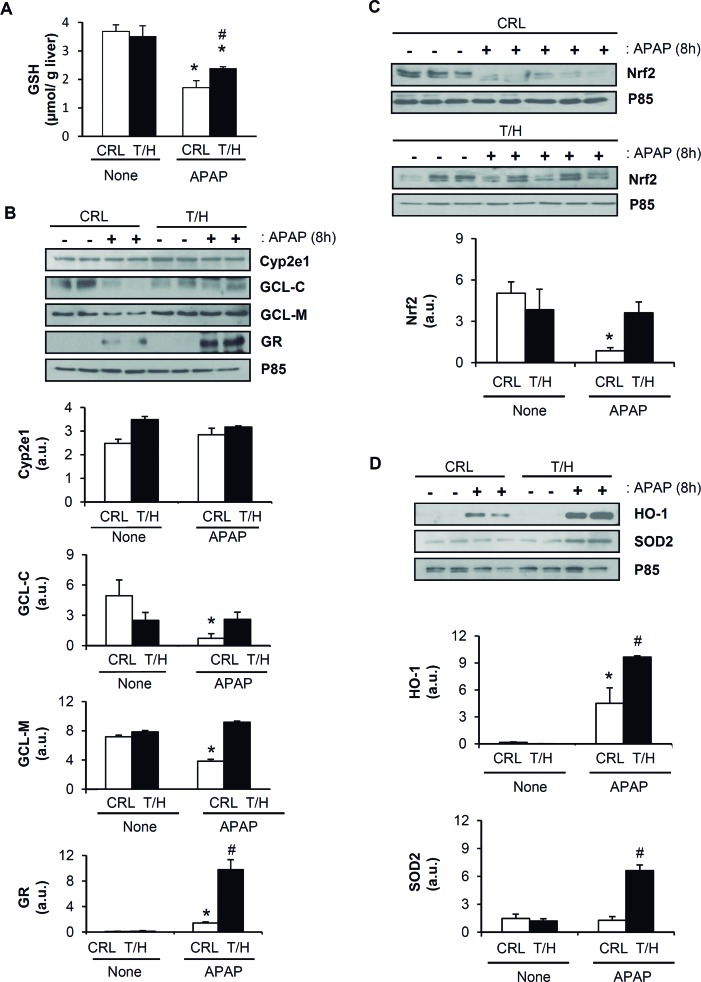
APAP treatment triggers a strong antioxidant response in the liver of hIF1 mice Control (CRL) and Tet-Off (T/H) mice were treated (+) or not (−) with APAP. The results shown are the mean ± S.E.M of 6 mice per group. **A**. Liver GSH content. **B**.-**D**. Blots of Cyp2e1, GCL-C, GCL-M, GR, Nrf2, HO-1, SOD2 and P85 (used as loading control) in mice treated or not with APAP. Histograms show the quantification of the proteins normalized to p85 expression (arbitrary units, a.u.). *, *p* < 0.05 when compared to CRL non-treated (−) by Student's t test. #, *p* < 0.05 when compared to CRL-APAP treated by Student's *t* test.

In livers of both mouse phenotypes, GSH levels (Figure 8A) and the basal expression of cytochrome P450 (Cyp2e1) (Figure 8B), both the catalytic and the modifier subunits of γ-glutamyl cysteine ligase (GCL-C and GCL-M) (Figure 8B) and basal glutathione reductase (GR) levels (Figure 8B) were comparable. APAP administration decreased both GCL-C and GCL-M (Figure 8B) and partially depleted GSH (Figure 8A) at 8 h, these effects being significantly ameliorated in T/H mice (Figure 8B and Figure 8A, respectively). Moreover, the induction of the detoxifying enzyme glutathione reductase (GR) in response to APAP challenge was more pronounced in hIF1 expressing mice (Figure 8B).

As intracellular redox state is chiefly regulated by the transcription factor Nrf2, we examined the expression level of total Nrf2. Although no differences were found at the basal state (Figure 8C), APAP administration significantly increased total Nrf2 degradation in wild-type mice whereas it was preserved in livers from T/H mice (Figure 8C). Likewise, the induction of Nrf2 target genes hemoxigenase-1 (HO-1) and superoxide dismutase 2 (SOD2) in response to APAP was significantly higher in mice expressing hIF1 (Figure 8D), consistent with the lower oxidative damage of liver proteins found in these animals (Figure 6D). Furthermore, we investigated the expression of Nrf2 and of redox-related genes (GCL-C, GCL-M, GR and SOD2) in normal and tumor tissue of control and T/H mice (Supplemental Figure S7). The results obtained reveal that tumors from T/H mice have higher expression of Nrf2, γ-glutamyl cysteine ligase (GCL-M) and mitochondrial superoxide dismutase (SOD2) than tumors originated in control animals further supporting a role for hIF1in counteracting an Nrf2-guided stress response.

**Figure 9:**
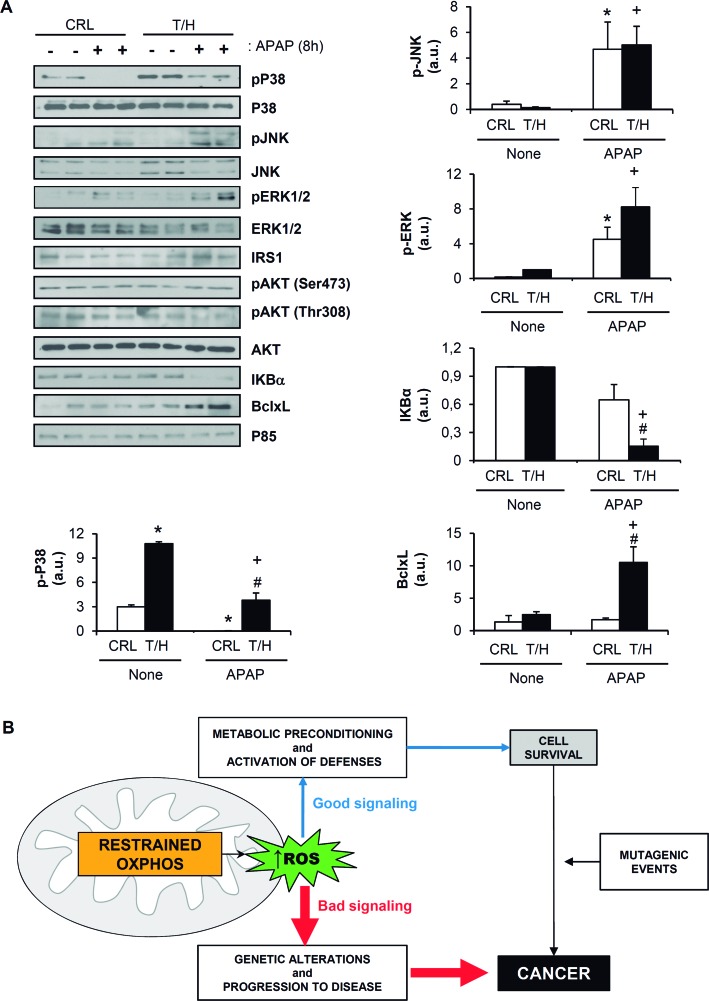
Signaling pathway in hIF1 expressing mice Control (CRL) and Tet-Off (T/H) mice were treated (+) or not (−) with APAP. The results shown are the mean ± S.E.M of 6 mice per group. **A**. Blots of the serine-threonine protein kinases: pP38 MAPK / P38 MAPK; pJNK / JNK; pERK1/2 / ERK 1/2 and pAKT / AKT. Expression of the NFκB inhibitor IκBα, and Bcl-xL in livers is also shown. P85 is shown as loading control. Histograms show the quantification of the proteins normalized to p85 expression (arbitrary units, a.u.). *, *p* < 0.05 when compared to CRL non-treated (−); #, *p* < 0.05 when compared to CRL-APAP treated (+) and +, *p* < 0.05 when compared to T/H non-treated (−) by Student's t test. **B**. The bad and the good of mitochondrial ROS. Inhibition of OXPHOS could trigger a rise in the levels of ROS. Mild ROS levels (good, in blue) signal metabolic preconditioning and the activation of defenses (mitohormesis) [59] that result in cell survival. On the contrary, high ROS levels (bad, in red) cause intracellular damage, genetic alterations and progression to disease. Mitohormesis is beneficial for certain type of stressing stimuli, but it can be detrimental in the case of mutagenic events that lead to cancer.

It has been demonstrated that CO, the product of HO-1, is able to inhibit tumor necrosis factor alpha (TNFα)–induced apoptosis in endothelial cells through the activation of p38 MAPK [37]. In light of the low HO-1 induction upon APAP injection in wild-type mice, phosphorylated p38 MAPK was barely detected in these animals (Figure 9A). By contrast, the basal phosphorylated p38 MAPK was significantly increased in livers of T/H mice (Figure 9A), reinforcing the metabolic stress of the hepatocytes in hIF1 expressing mice (Figure 1 and Supplemental Figures S1 and S2). Moreover, and in contrast with wild type mice, T/H mice maintained phosphorylated p38 MAPK 8h after APAP injection (Figure 9A). The expression and phosphorylation of other stress kinases such as JNK and ERK 1/2 was not significantly different between control and T/H mice (Figure 9A). No differences were also found in survival signaling pathways mediated by IRS1/Akt between control and hIF1 expressing mice (Figure 9A). Remarkably, APAP-treated hIF1-expressing mice showed almost complete degradation of the NFκB repressor IκBα when compared to control treated mice (Figure 9A), supporting also the activation of this survival pathway [38]. Consistently, the anti-apoptotic Bcl-xL protein was increased at 8h post-APAP injection exclusively in T/H mice (Figure 9A).

## DISCUSSION

Herein, we demonstrate *in vivo* that the expression of an active mutant of hIF1 in hepatocytes partially inhibits OXPHOS. The inhibition of OXPHOS results in a partial energy deficit in the liver, the activation of the metabolic stress kinase AMPK and a limited gluconeogenesis after a mild overnight fast of the animals. Inhibition of OXPHOS by hIF1 is exerted both by limiting the respiratory rate at the level of Complex IV and by inhibiting the activity of the ATP synthase. The inhibition of the activities of these complexes correlated with alterations in both the assembly and activities of super-complexes [39]. Within this background, a hIF1-limited activity of OXPHOS in the liver predisposes to cancer progression. Mechanistically, it appears that an enhanced cancer progression in hIF1-expressing mice is related to its pro-oncogenic activity that favors an increased proliferation and an increased resistance to execute apoptosis by the hepatocarcinomas. Inhibition of cell death seems to be unrelated to hIF1-mediated differences in PTP opening. Moreover, it appears that a restrained OXPHOS switches on a stress response that is most likely mediated by the cellular energy and redox states of the liver that triggers the basal activation of AMPK and p38 MAPK. This is particularly evident when the animals are challenged by an APAP overdose that induces a strong redox Nrf2 mediated response that involves the activation of the NFκB pathway and the overexpression of Bcl-xL favoring cell survival. It is within this metabolic background when genetic alterations that lead to carcinogenesis are able to progress because of a limited capacity to execute cell death by mitochondrial activity (Figure 9B), in agreement with recent suggestions [5] and the clinical observations that cancer recurrence is linked to the bioenergetic dysfunction of mitochondria (for review [1]).

Recent findings in *in vivo* mouse models indicate that the activation of mitochondrial OXPHOS provides a tumor-suppressive metabolic state [5, 40]. However, the role of the partial suppression of OXPHOS in cancer onset and progression has not been addressed so far. The availability of the Tet-Off T/H mice afforded a unique opportunity to verify *in vivo* the role of OXPHOS in carcinogenesis. Although T/H mice do not develop spontaneous tumors after more than one year follow up, perhaps because they overexpressed p38 MAPK which is a known tumor suppressor in the liver [41], they generated a three-fold higher number of carcinomas than control mice when exposed to DEN, supporting an oncogenic role for IF1 and a tumor-suppressive role for an enhanced activity of OXPHOS in the liver.

We have previously suggested a role for IF1 in carcinogenesis [14, 18]. Recent findings have stressed that a high expression level of IF1 is a bad predictor of survival and recurrence of the disease in liver, bladder and gastric cancer patients [19, 42, 43]. Moreover, IF1 promotes tumorigenesis and metastasis of human hepatocarcinomas and gastric cancer cells as assayed in mouse xenografts [19, 42]. In liver cancer, IF1 propitiates metastasis and angiogenesis through non-canonical signaling of the NFκB pathway to Snai1, which mediates epithelial mesenchymal transition, and to VEGF, that induces angiogenesis [19]. In bladder cancer, the oncogenic role of IF1 seems to be dependent on the stimulation of proliferation by cyclins and cyclin-dependent kinases related to the G1/S transition of the cell cycle [43]. In gastric cancer [42], similarly to what we observed in colon cancer [14], the oncogenic role of IF1 implicates an enhanced proliferation and cell-death resistant phenotype. Cell death resistance in colon and lung cancer is mediated by ROS-signaling to the canonical NFκB pathway [14, 18], whereas in breast and ovarian cancer the mechanism linking IF1 to the cell-death resistant phenotype remains to be investigated [18]. Hence, our findings in the Tet-Off T/H mice provide the first *in vivo* demonstration for the oncogenic role of IF1 that results from an enhanced proliferation and cell-death resistant phenotype of the hepatocyte, the latter attained by metabolic pre-conditioning of the anti-oxidant response of the liver.

It should be noted that carcinogenesis differentially affects the expression of IF1 in a tissue specific manner [18]. Moreover, the overexpression of IF1in the carcinomas not always correlate with a bad patient prognosis [19, 42, 43]. In fact, the overexpression of IF1 in breast and colon cancer predicts much better patient's prognosis [18]. These differences might stem from the different metabolic traits of the normal tissues as reveal by the large tissue-specific differences in the expression of IF1, which is very high in liver, kidney and stomach and almost negligible in breast, colon and lung [18]. At present time, we do not have a mechanistic explanation for the apparent tissue-specific differences in the oncogenic behavior of IF1. We speculate that mechanisms that involve a differential susceptibility of the different cell types to particular forms of stress and/or recognition by the immune system could be at the bases of these differences.

Interestingly, Dox administration that is used to silence the hIF1 transgene, and is also known to inhibit the growth of different cancer cells [20], promoted significant reductions in DEN-induced carcinogenesis both in the livers of control and hIF1 expressing mice, strongly supporting the use of this antibiotic as an anticancer agent [44, 45] and further emphasizing that targeting mitochondria translation offers an additional strategy to combat the disease.

Chronic liver injury is a major risk factor for the development of hepatocellular carcinomas. Interestingly, it has been recently demonstrated that dysfunction of mitochondrial oxidative phosphorylation ensues during the early stages of cirrhosis propitiating an enhanced rate of glycolysis [46] which is the metabolic pathway that chiefly provides the metabolic precursors that sustain proliferation of carcinomas [1]. In agreement with the role played by IF1 as inhibitor of OXPHOS and stimulator of both glycolysis [15, 18] and proliferation of cancer cells [14, 18, 19] we have observed that hIF1 expression in the liver mediates the rewiring of metabolism to an enhanced glycolysis that could support the elevated proliferation rate of the carcinomas. Moreover, the enhanced carcinogenesis observed in the liver of hIF1 expressing mice is also contributed by the reduced apoptosis observed in these tumors. Inhibition of the H^+^-ATP synthase by hIF1 is known to generate a mild ROS signal that induces NFκB-guided nuclear programs of cell death evasion [8, 14, 18] and activate epithelial-mesenchymal transitions that favors metastasis [19]. This redox condition of the liver is consistent with the pathophysiological context for the development of hepatocellular carcinomas. Indeed, it has been previously suggested that increased intracellular ROS in chronic inflammatory states of the liver protect against cell death [47] and the increased basal ROS levels of cirrhotic hepatocytes seem to determine its increased apoptosis resistance [48]. Altogether, these findings reinforce the crucial role played by mitochondrial dysfunction during tumor transformation and suggest that the inhibition of the H^+^-ATP synthase could represent a primary event in hepatic injury leading to the onset of carcinomas.

Energy limitation by a restrained activity of OXPHOS is known to induce the metabolic stress kinase AMPK [49] and to generate a mitochondrial ROS signal of mild intensity [14]. This signal could also contribute to the induction of AMPK and of the stress p38 MAP kinase since it could modify the reduction state of thioredoxin which is known to halt the activation of both AMPK [50] and p38 MAPK [51]. The redox-mediated preconditioning is manifested when the animals are challenged with a ROS stressor such as APAP. Hence, we support that ROS-mediated signaling [8, 14] is the primary mechanism involved in evasion of cell-death mediated by IF1 in the liver. Indeed, the maintenance of total Nrf2 expression in the liver of T/H mice orchestrates a stress response that involves the overexpression of SOD2 and HO-1 as well as the detoxification of oxidized glutathione to provide protection against oxidative stress and cell death. Metabolic preconditioning and protection against death seems to be exerted by the basal activation of p38 MAPK and AMPK because other stress kinases such as JNK, ERK1/2 and Akt are not modified by the overexpression of hIF1 in the liver. The transcription factor NFκB is a crucial regulator of cell survival pathways [52]. The degradation of IKBα, which is the repressor of the NFκB pathway, and the concurrent accumulation of the anti-apoptotic Bcl-xL in the liver of T/H mice once again highlight the crosstalk between ROS and NFκB, and further implicate this pathway in hIF1-mediated cell survival after APAP treatment. Moreover, the transcriptional crosstalk between IF1 and the NFκB pathway has also been recently implicated in other hallmarks of liver cancer such as promoting angiogenesis and metastasis [19]. We cannot exclude that the maintenance of the phosphorylated state of p38 MAPK after APAP-induced cell death in T/H mice could contribute to cell survival because it has been shown to suppress ROS accumulation and cell death in hepatocytes [41]. Moreover, the activation of p38 MAPK has been reported to inhibit TNFα-induced apoptosis in endothelial cells [37]. In any case, the activation of different signaling pathways upon OXPHOS-mediated tissue preconditioning, p38 MAPK in the liver (this study) and PI3K/Akt in neurons [8], supports a large cell-type variability in mitochondrial signaling responses.

Overall, our data provide the first *in vivo* demonstration that the partial inhibition of OXPHOS generates a metabolic phenotype that is more prone to the development of cancer (Figure 9B), further indicating the pro-oncogenic nature of IF1. Hence, we conclude that the bioenergetic activity of mitochondria acts as a tumor-suppressor and guardian of cancer onset and progression.

## MATERIALS AND METHODS

### Transgenic animals

Transgenic mice containing the mutant H49K version of hIF1 (TRE-H49K-25, H) were obtained by pronuclear microinjection [8]. The commercially available B6.Cg-Tg(Lap-tTA)5Bjd/J mice (T) and B6.Cg-Tg(Cewbpb-rtTA2S*S2)1Bjd/Ibcm mice (rT), expressing respectively the transactivators tTA or rtTA in hepatocytes were used. The Tet-Off and Tet-On (T/H) double transgenic animals were obtained. To express hIF1, double transgenic T/H mice were administered (Tet-On) or not (Tet-Off) Dox (2mg/mL) in the drinking water. Animal experiments were carried out after approval of the Institutional Review Board (CEI-52-961) in compliance with animal policies and ethical guidelines of the European Community.

### Determination of blood and liver metabolites

Adenine nucleotides and other liver metabolites were extracted from frozen liver powder with a 6% perchloric acid. The ATP concentration was determined using the ATP Bioluminescence Assay Kit CLS II (Roche). Liver GSH and GSSG concentrations were determined using the Glutathione Assay Kit (BioVision Inc.).

### Western blotting, antibodies and protein carbonylation

The primary monoclonal antibodies developed in our lab and used in this study were: HSP60 (1:5,000), anti-NADH-9 (1:1,000), anti-β-F1-ATPase (1:20,000), anti-LDH-A (1:1,000) and anti-GAPDH (1:20,000) [53, 54]. The monoclonal antibody specifically recognizing the human [15] and mouse (Molecular Probes) IF1 proteins were used at 1:200 dilution. Other antibodies used were: anti-SDH-B (1:1000) from Life Technologies; anti-Complex III subunit Core 2 (1:1,000), anti-COXI (1:1,000), anti-COXIV (1:1,000) and anti-Cyp2e1 antibody (aB19140) from Abcam; anti-SOD2 (1:5,000), anti-JNK (sc-571; 1:1000), anti-phospho-Akt (Ser473) (sc-7985-R; 1:1000), anti-phospho-Akt (Thr308) (sc-16646-R; 1:1000) and anti-Nrf2 (C-20), (sc-722; 1:1000) from Santa Cruz Biotechnology, Inc.; anti-Bclx (610211; 1:1000) antibody from BD Biosciences; anti-IκBα (1:1,000), anti-phospho JNK (Thr183/Tyr185) (#4668, 1:1000), anti-phospho p38 MAPK (Thr180/Tyr182) (#9211; 1:1000), anti-p38 MAPK (#9212; 1:1000), anti-Phospho-p53(Ser15) (1:400) and anti-Akt (#9272; 1:1000) antibodies from Cell Signaling Technology Inc; anti-IRS1 (06-248; 1:1000), anti-p85 (06-195; 1:1000) and anti-HO1 (AB1284; 1:1000) antibodies from Upstate (Millipore); Anti-Ki-67 (SP6) (RM-9106-S1 1:200) from Fischer Scientific; Anti-F4/80 (123101; 1:200) from BioLegend; Anti-GCLc and GCLm antibodies were a gift from T Kavanagh (University of Washington, USA). Peroxidase-conjugated anti-mouse, anti-goat or anti-rabbit IgGs (Nordic Immunology; 1:3,000) and Biotin-SP anti-rat IgG (712-065-153; 1:500) from Jackson ImmunoResearch Inc. were used as secondary antibodies. For the determination of protein carbonylation, the Oxyblot Oxidized Protein Detection kit (Chemicon International Inc.) was used. The blots were revealed using the ECL^®^ reagent (Amersham Pharmacia Biotech). The intensity of the bands was quantified using a Kodak DC120 digital camera and the Kodak 1D Analysis Software.

### Mitochondrial studies

Mitochondrial respiration was measured with the use of a Clark-type electrode [8]. The spectrophotometric determination of the activity of OXPHOS complexes was also carried out [55]. For Blue native (BN)-PAGE, Clear native (CN)-PAGE and In-gel activities, liver mitochondria were solubilized at the indicated digitonin/protein ratios [56].

### Reverse phase-liquid chromatography RP-LC-MS/MS analysis (Dynamic Exclusion Mode)

After drying, gel bands from BN-PAGE were digested *in situ* with 12.5 ng/μl of trypsin (Promega) in 50 mM ammonium bicarbonate pH 8.8. Digestion was stopped by the addition of 1% trifluoracetic acid. Supernatants were dried down and then desalted onto ZipTip C18 Pipette tips (Millipore). The desalted protein digest was dried, resuspended in 10 μl of 0.1% formic acid and analyzed by RP-LC-MS/MS in an Easy-nLC II system coupled to an ion trap LTQ-Orbitrap-Velos-Pro mass spectrometer (Thermo Fischer Scientific, Bremen, Germany). The peptides were concentrated (on-line) by reverse phase chromatography using a 0.1mm×20 mm precolumn Acclaim PepMap C18, 5 μm, 100 A (Thermo Fischer Scientific), and then separated using a 0.075mm x 150 mm column Acclaim PepMap C18, 3 μm, 100 A (Thermo Fischer Scientific) operating at 0.3 μl/min. Peptides were eluted using a 90-min gradient from 5 to 40% solvent B (solvent A: 0.1% formic acid in water, solvent B: 0.1% formic acid, 80% acetonitrile in water). ESI ionization was done using a Nano-bore emitters Stainless Steel ID 30 μm (Proxeon) interface. The Orbitrap resolution was set at 30,000. Peptides were detected in survey scans from 400 to 1600 amu (1 μscan), followed by fifteen data dependent MS/MS scans (Top 15), using an isolation width of 2μ (in mass-to-charge ratio units), normalized collision energy of 35%, and dynamic exclusion applied during 30 seconds periods. Peptide identification from raw data was carried out using the SEQUEST algorithm (Proteome Discoverer 1.3, Thermo Scientific). Database search was performed against uniprot-mus.fasta. The following constraints were used for the searches: tryptic cleavage after Arg and Lys, up to two missed cleavage sites, and tolerances of 10 ppm for precursor ions and 0.8 Da for MS/MS fragment ions and the searches were performed allowing optional Met oxidation and Cys carbamidomethylation. Search against decoy database (integrated decoy approach) using false discovery rate (FDR) < 0.01.

### Carcinogenesis

For an exploratory experiment of DEN-induced carcinogenesis two-week old control and Tet-Off T/H male mice received or not a single i.p. injection of DEN (25 mg/kg bw) [23] and the animals were sacrificed at week 44. In the main experiment, two-week old control (n=35) and Tet-Off T/H (n=35) mice received or not an i.p. injection of DEN (0.33 mmol/kg bw) once weekly for a total of 26 weeks [24]. Liver glutamate/pyruvate transaminase (GPT) was evaluated every 2 weeks using the Reflotron plus analyzer (Roche Diagnostics, Penzberg, Germany). Mice were sacrificed at week 29.

### Hepatotoxicity

Six month old control (n=8) and Tet-Off T/H (n=8) mice were used to induce hepatotoxicity. The animals were fasted for 14 hours and then received a single i.p. injection of APAP (300 mg/kg; Sigma Aldrich). Eight hours after the injection mice were sacrificed.

### Immunohistochemistry and immunofluorescence microscopy

The peroxidase based EnVisionTM FLEX Mini kit High pH (Dako Cytomation) and VECTASTAIN ABC kit (Vector Laboratories, Inc.) were used for immunohistochemistry using primary antibodies listed in Western blotting, antibodies and protein carbonylation section. The TUNEL kit (Roche) and anti-activated caspase 3 (1:250) were used to assess the rate of cell death. Secondary Cy-3/Cy-5-conjugated antibodies were used (Millipore Bioscience Research Reagents). Cellular fluorescence was analyzed by confocal microscopy.

### Electron microscopy

Liver slices were fixed and embedded in Epon (TAAB 812 resin; TAAB Laboratories Equipment, Aldermaston, Berks, UK). Ultrathin sections were stained and processed for electron microscopy as described [26].

### Quantitative RT-PCR analysis

RNA samples were extracted using the RNAeasy mini kit (QIAGEN). Reverse transcription (RT) reactions were performed using 1μg of total RNA and the High Capacity Reverse Transcription Kit (Applied Biosystems) with random primers. Primers were designed with Probe Finder Software (Roche Applied Science). The primers used were: m-IF1-F: agaagctggtggagccttc, m-IF1-R: ggcagccagctgttctttag; m-β-F1-ATPase-F: ggcacaatgcaggaaagg, m-β-F1-ATPase-R: tcagcaggcacatagatagcc; m-Hsp60-F: tcttcaggttgtggcagtca, m-Hsp60-R: cccctcttctccaaacactg; m-GAPDH-F: agcttgtcatcaacgggaag, m-GAPDH-R: tttgatgttagtggggtctcg; m-PGC1α-F: gaaagggccaaacagagaga, m-PGC1α-R: gtaaatcacacggcgctctt; m-β-actin-F: ctaaggccaaccgtgaaaag, m-β-actin-R: accagaggcatacagggaca; h-IF1-F: gggccttcggaaagagag, h-IF1-R: ttcaaagctgccagttgttc. Real-time PCR was performed using Power Sybr Green PCR Master Mix (Bio-Rad) and an ABI PRISM 7900 SDS thermocycler (Applied Biosystems). The relative expression of the mRNAs was determined using the comparative ΔΔCt method with β-actin as control and relative to the normal tissues.

### Determination of mitochondrial DNA (mtDNA) copy number

DNA (nDNA+mtDNA) was extracted using DNeasy Blood & Tissue Kit from Qiagen. Quantification of mtDNA (mtDNA/nDNA) was performed by qPCR in the ABI PRISM 7900HT SDS thermocycler (Applied Biosystems, Singapore) using 6 ng of DNA, 0.25 μM of primers and the Power Sybr Green PCR Master Mix (Bio-Rad). The nuclear ATPIF1 and the mitochondrial 12S rRNA encoded genes were chosen to determine the ratio of mtDNA to nDNA. The primers used were: m-12S-F: cctcttagggttggtaaatttcg, m-12S-R: cgaagataattagtttgggttaatcg; m-IF1-F: agaagctggtggagccttc, m-IF1-R: ggcagccagctgttctttag. The relative mtDNA copy number was calculated using the ΔΔCt method and the 12S/IF1 ratio used to compare the samples.

### Swelling and Ca^2+^ uptake in isolated mitochondria

The calcium retention capacity (CRC) of isolated mitochondria was measured with the Ca^2+^-sensitive fluorescent probe Calcium-Green 5N (0.1 μM, excitation 506 nm, emission 532 nm) as previously described [57], using an Aminco-Bowman II fluorimeter (SLM/Aminco, Urbana, IL, USA). Mitochondrial swelling was measured by monitoring the decrease in absorbance of the suspension at 540 nm [58], using a Nicolet Evolution 300 spectrophotometer (Thermo Scientific, Warrington, UK). All experiments were carried out at 37°C in the presence of 1 mM MgCl_2_, respiratory substrates (5 mM succinate + 2 μM rotenone) and in the presence or absence of 1 mM ATP-Mg or 5 μM CsA. After 3-5 min of incubation, mitochondria were challenged with subsequent 2.5-10 nmol CaCl_2_ additions as indicated in the figure legends, and Ca^2+^ uptake into mitochondria was measured as a decrease in fluorescence. For swelling assays, the absorbance at 540 nm of mitochondria was monitored through sequential additions of 100-500 nmol CaCl_2_.

### Statistical analysis

Statistical analyses were performed using a two-tailed Student's t-test. ANOVA with post hoc Dunnett's test were used for multiple comparisons to the control, using the SPSS 17.0 software package. The results shown are means ± SEM. A *p* < 0.05 was considered statistically significant.

## SUPPLEMENTARY MATERIAL TABLE AND FIGURES


