# Supplementary material for: Down-regulation of oxidative phosphorylation in the liver by expression of the ATPase inhibitory factor 1 induces a tumor-promoter metabolic state
Source: Oncotarget. 2015 Nov 22;7(1):490–508. doi: 10.18632/oncotarget.6357 (PMC4808013; doi:10.18632/oncotarget.6357)
Supplement: Supplementary file 1 [file oncotarget-07-0490-s001.pdf]

Down-regulation of oxidative phosphorylation in the liver by expression of the ATPase inhibitory factor 1 induces a tumor-promoter metabolic state

Supplemental Material

Supplemental Table S1, Related to Fig. 2. Protein bands labeled as $(CV)_2$, SC and FoF1 in BN-gels (see Fig. 2D) were trypsin digested and the peptides obtained analyzed by RP-LC-MS/MS. The known proteins of different respiratory complexes (CI to CV) are indicated. The number of proteins and peptides identified for each of the complexes in each band is shown. The percentage of proteins belonging to each complex is also indicated.

Band		C I		C II		C III		C IV		C V	
	Proteins	45		4		10		19		18	
		Number	%	Number	%	Number	%	Number	%	Number	%
$(CV)_2$	Proteins	11	24.4	--	--	2	20	1	5.3	6	33.3
	Peptides	38		--		10		3		20	
(SC)	Proteins	24	53.3	--	--	4	40	1	5.3	6	33.3
	Peptides	106		--		21		2		43	
(F_0F_1)	Proteins	--	--	--	--	1	10	3	15.8	10	52.6
	Peptides	--		--		4		7		79	

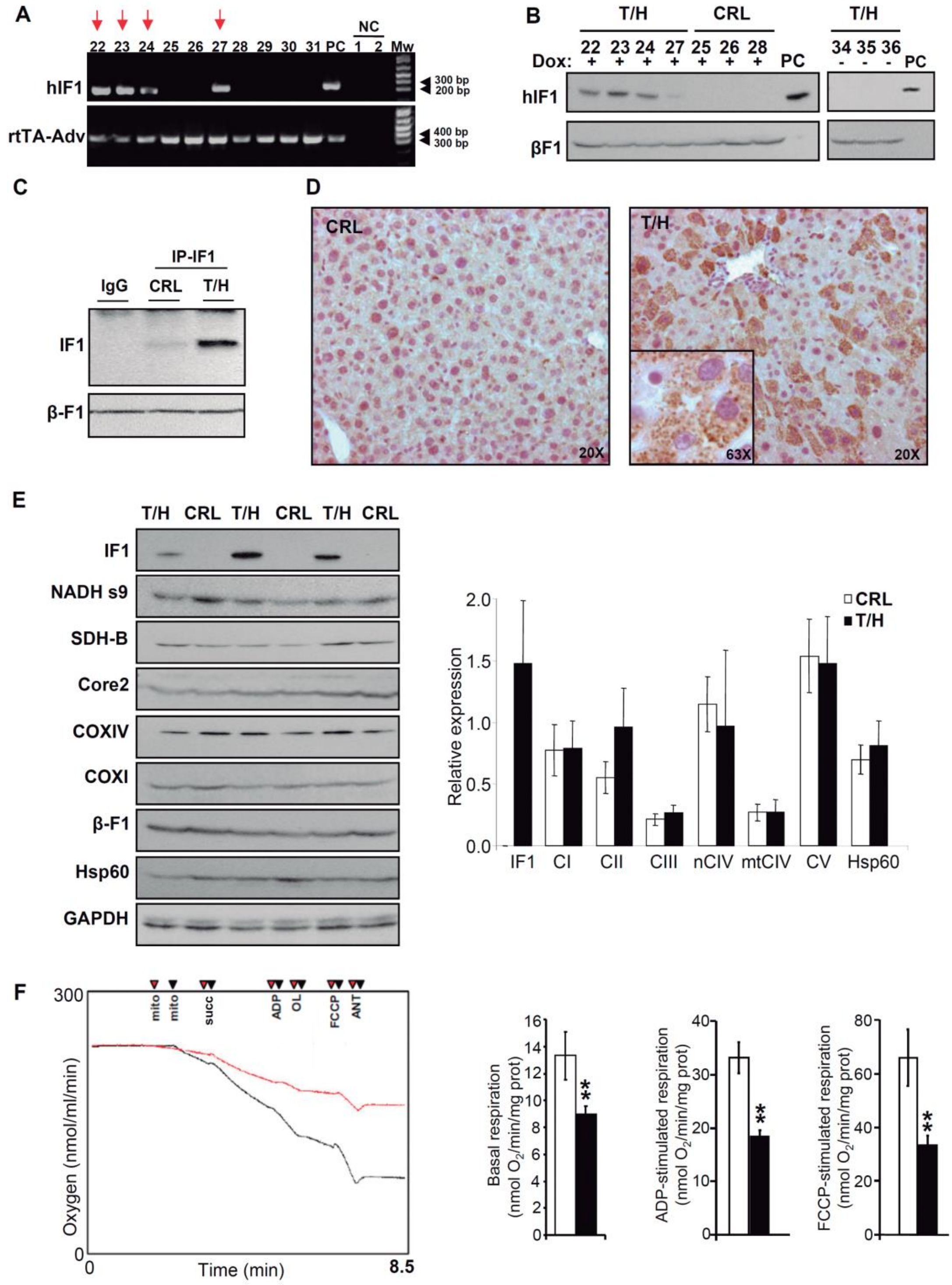

Figure S1, Related to Fig. 1. OXPHOS is inhibited in the liver of Tet-On mice expressing hIF1. (A) PCR analysis for the H49K variant of human IF1 (hIF1) and rtTA-Adv constructs. Red arrows indicate double-transgenic mice. (**B**) Western blots show the expression of hIF1 (12 kDa) in double transgenic (T/H) and control (CRL)

mice (identified by numbers) in the presence (+) or absence (-) of doxycycline (Dox). β-F1-ATPase (β-F1) expression is shown as a loading control. In **A** and **B**, PC and NC, positive and negative controls, respectively. (**C)** Liver extracts from CRL and T/H mice were subjected to immunoprecipitation (IP) using a monoclonal anti-IF1 antibody and subsequently analyzed by western blotting. Non-immune IgG is used as negative control. (**D**) Immunohistochemistry for hIF1 in the liver of CRL and T/H mice. Magnification 20X and 63X (inset). (**E**) Representative Western blots of the expression of hIF1, mitochondrial complex I (NADH s9), complex II (SDH-B), complex III (Core2), complex IV (COX I and COX IV) and complex V (β-F1), heat-shock protein 60 (Hsp60) and the glycolytic GAPDH in liver extracts of CRL and T/H mice. Three different mice per condition tested are shown. Histograms to the right show the relative expression of each protein in CRL and T/H mice. (**F**) Polarographic profiles of isolated mitochondria from CRL (black trace) and T/H mice (red trace). The effect of succinate, ADP, oligomycin (OL), FCCP, and antimycin A (ANT) is shown. Histograms show the basal, ADP-stimulated and FCCP-stimulated respiration in mitochondria of T/H mice (closed bars) when compared to CRL (open bars). Bars are the mean ± SEM of 5 CRL and 7 T/H animals. *, $p<0.05$ when compared to control mice by Student's t test.

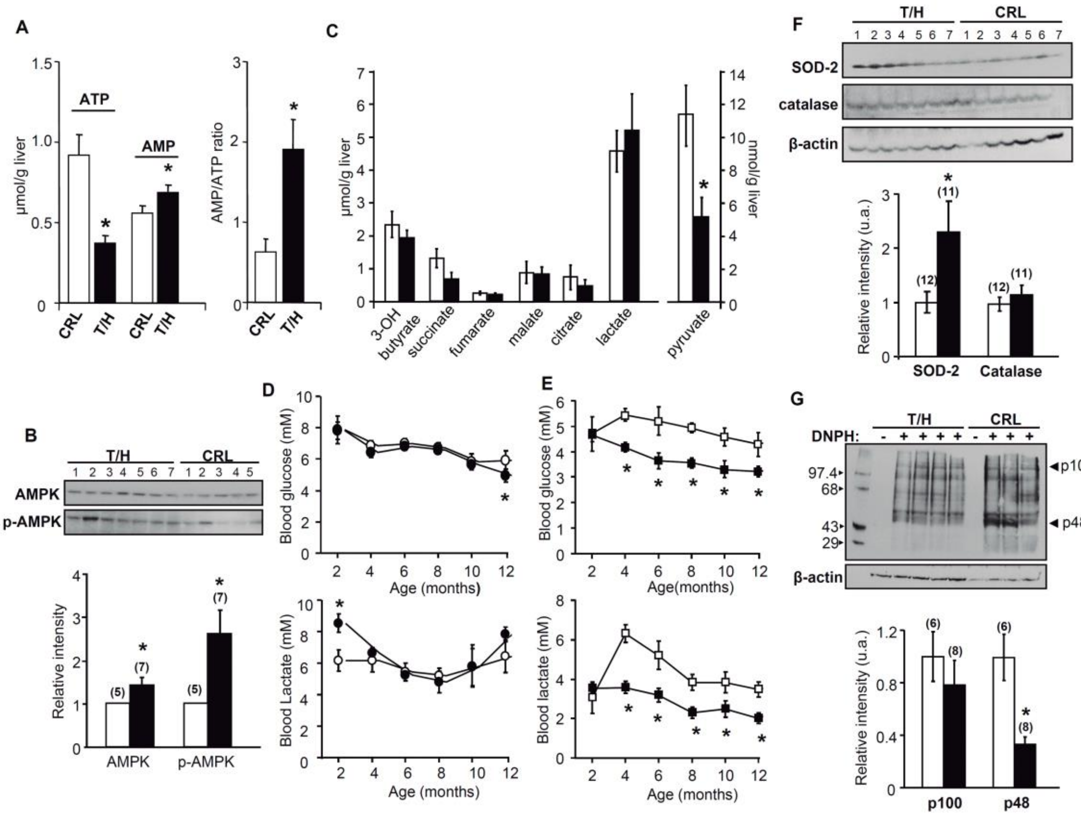

Figure S2, related to Fig. 1. Expression of hIF1 compromises the liver energy state. **(A)** Content of adenine nucleotides (ATP and AMP) and the AMP/ATP ratio in liver extracts of control (CRL, open bars) and T/H mice (closed bars). Histograms show the mean ± SEM of 12 mice per group. (**B)** Western blots show the hIF1-dependent phosphorylation of AMPK (p-AMPK) in liver extracts. Histograms show the mean ± SEM of 5 CRL and 7 T/H mice. (**C)** Liver organic acids. Histograms show the mean ± SEM of 4 CRL and 6 T/H mice. (**D** and **E**) Blood glucose and lactate concentrations in CRL (open dots and squares) and T/H (closed dots and squares) mice under fed (D) or fasted (E) conditions. The results shown are the mean ± SEM of 6 mice per group. (**F**) Expression of superoxide dismutase 2 (SOD2) and catalase in the liver of CRL and T/H mice. β-actin is shown as loading control. The histograms show the mean ± SEM of 12 and 11 mice for CRL and T/H mice, respectively. (**G**) Representative experiment showing the differential carbonylation of liver proteins in CRL and T/H mice. The

presence (+) or absence (-) of DNPH is indicated. Protein loading of the samples was verified by western blotting with anti β-actin. The migration of molecular mass markers is indicated to the left. Arrows (to the right) identify the migration of the two proteins used in quantification of protein carbonylation in the histograms. The results shown are the mean ± SEM of 6 CRL (open bars) and 8 T/H (closed bars) mice, respectively. (**A-G**) *, $p<0.05$ when compared to control mice by Student's t test.

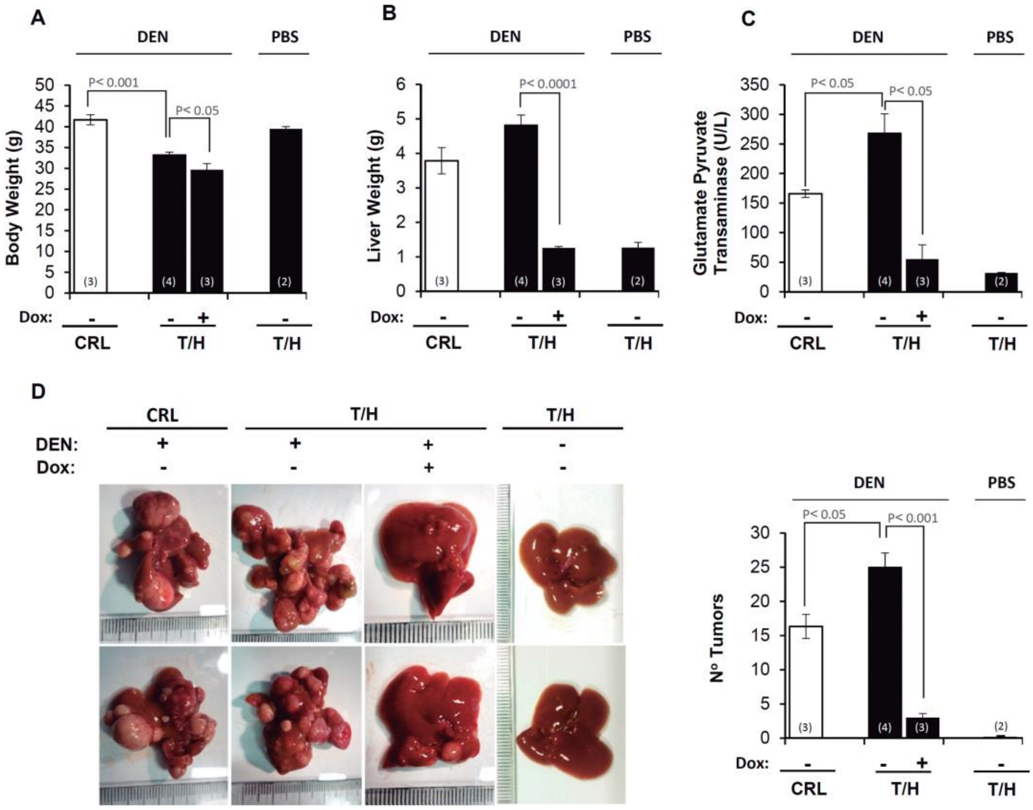

Figure S3, related to Fig. 3. The expression of hIF1 increased DEN-induced hepatocarcinogenesis in the single DEN-administration model. (A-C) Changes in body weight (**A**), liver weight (**B**) and blood GPT (**C**) in PBS or DEN-treated CRL (open bars) and hIF1 Tet-Off T/H (closed bars) mice, treated in presence (+) or absence (-) of Dox. **(D)** CRL and T/H livers of mice treated (+) or not treated (-) with DEN. The effect of Dox administration (+) or not (-) is also shown. The number of tumors in PBS or DEN-treated CRL (open bars) and T/H (closed bars) mouse livers treated (+) or not (-) with Dox. The number of mice is indicated in parenthesis. Results are means ± SEM. P values by Student's t test are indicated.

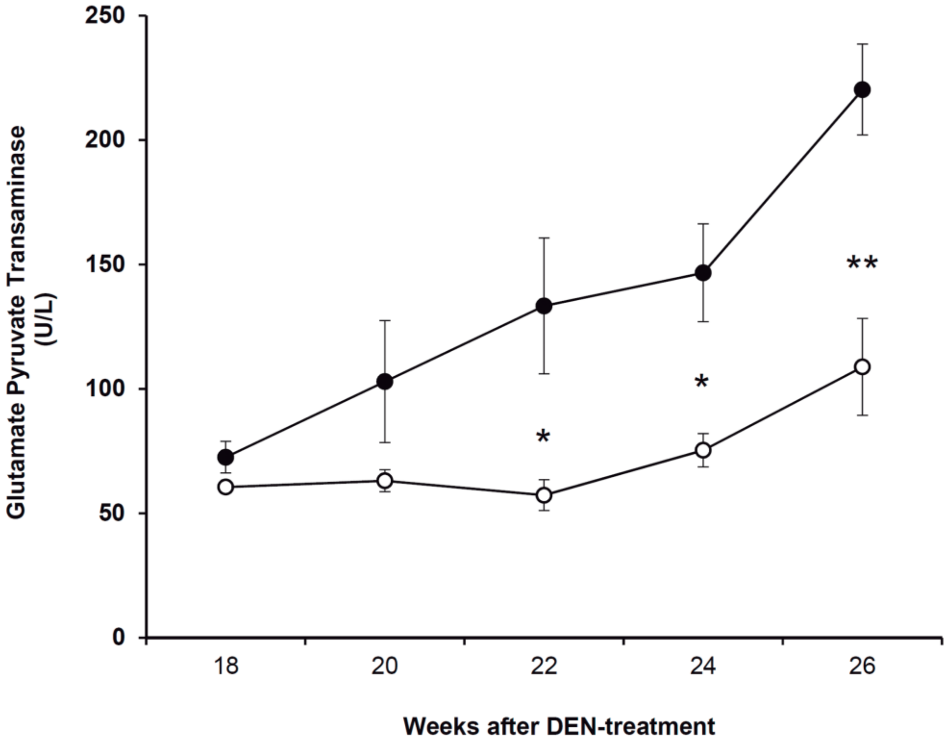

Figure S4, related to Fig.3. Progression of blood GPT levels during DEN-induced hepatocarcinogeneis. Blood glutamate/pyruvate transaminase activity (U/l) was determined in DEN-treated control (CRL, open circles) and hIF1 Tet-Off T/H (closed circles) mice. The results are means ± SEM of eleven animals per group. *, $p<0.05$ and **, $p<0.01$ when compared to control mice by Student’s t test.

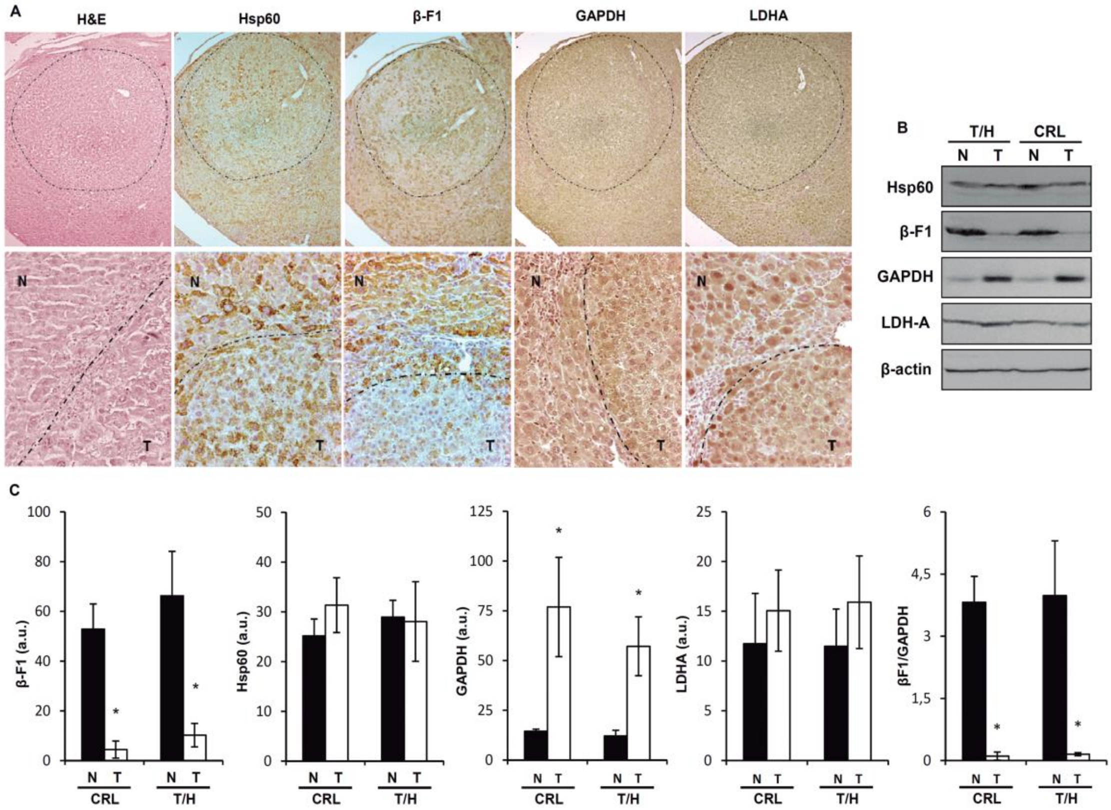

Figure S5, Related to Fig. 5. Repression of mitochondrial biogenesis in DEN-induced hepatocarcinogenesis (HCC). (A) Focal hepatic lesions in DEN-treated CRL mice identified by hematoxilin-eosin (H&E) and immunohistochemical staining with the antibodies against Hsp60, β-F1-ATPase (β-F1), GAPDH and LDHA in the same localized tumor area (upper panels, magnification 5x). The same study in normal (N, upper left) and tumor (T, lower right) areas at 20x magnification (lower panels). A dashed line marks the border between tumor and non-tumor areas**. (B)** Blots of Hsp60, β-F1-ATPase (β-F1), GAPDH, LDHA and β-actin (loading control) in normal (N) and tumor (T) tissue from CRL and T/H mice. (**C**) Histograms show the quantification of the proteins normalized to β-actin expression (arbitrary units, a.u.). The bioenergetic signature (β-F1-ATPase/GAPDH ratio) of the tissues is also shown. Results are means ± SEM for 7 animals. *$P < 0.05$ when compared with normal by Student's t test.

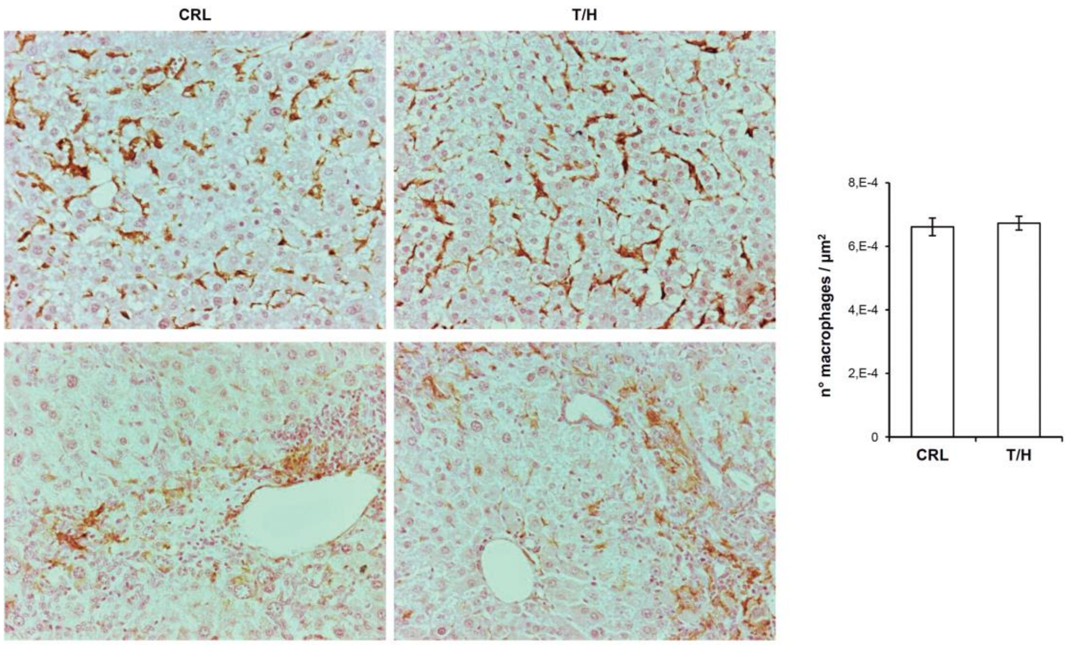

Figure S6, Related to Fig. 5. Macrophage infiltration in DEN-induced hepatocarcinogenesis (HCC). (A) Liver sections of DEN-treated CRL and hIF1 Tet-Off T/H mice were processed for immunohistochemistry to assess the rates of macrophage infiltration in HCC. F4/80 staining is shown in tumor (upper panels) and normal (lower panels) tissues. Magnification 20x. Histograms show the quantification of macrophage infiltration (number of macrophages/μm^2) in the tumor area from CRL and T/H animals. Bars are the mean ± SEM of 4 mice per group.

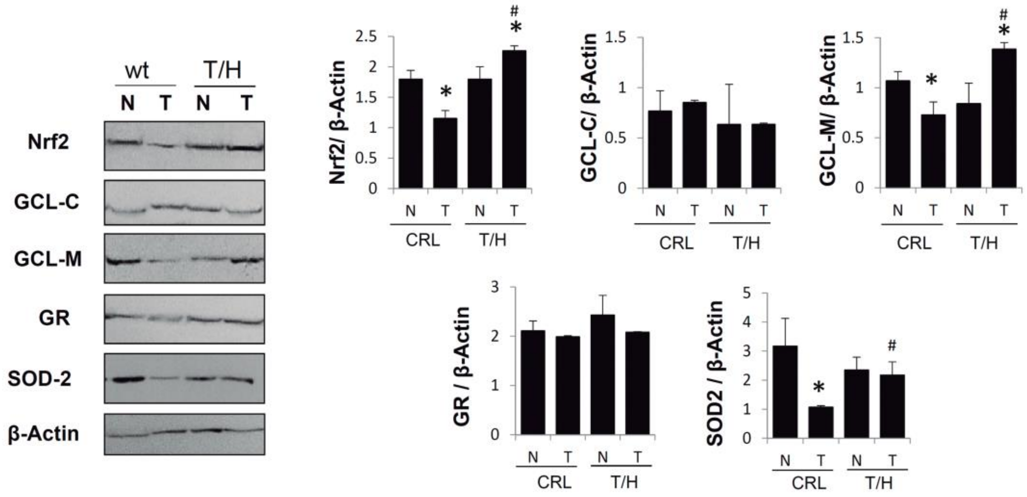

Figure S7, Related to Fig. 8. DEN treatment triggers an antioxidant response in the liver of hIF1 mice. Normal (N) and tumor (T) tissues from control (CRL) and Tet-Off T/H mice were analyzed after treatment with DEN. Blots of Nrf2, GCL-C, GCL-M, GR, SOD2 and β-Actin (loading control) are shown. Histograms show the quantification of the proteins normalized to the β-actin signal. The results are the mean ± S.E.M of 4 mice per group.*, $p<0.05$ when compared to normal tissue by Student's t test. #, $p<0.05$ when compared to tumor tissue in CRL by Student's t test.
